# Ring trial of 2nd generation RT‐QuIC diagnostic tests for sporadic CJD

**DOI:** 10.1002/acn3.51219

**Published:** 2020-11-13

**Authors:** Christina D. Orrú, Bradley R. Groveman, Aaron Foutz, Matilde Bongianni, Franco Cardone, Neil McKenzie, Audrey Culeux, Anna Poleggi, Katarina Grznarova, Daniela Perra, Michele Fiorini, Xiaoqin Liu, Anna Ladogana, Marco Sbriccoli, Andrew G. Hughson, Stéphane Haïk, Alison J. Green, Michael D. Geschwind, Maurizio Pocchiari, Jiri G. Safar, Gianluigi Zanusso, Byron Caughey

**Affiliations:** ^1^ Laboratory of Persistent Viral Diseases Rocky Mountain Laboratories National Institute for Allergy and Infectious Diseases National Institutes of Health Hamilton Montana USA; ^2^ Departments of Pathology and Neurology Case Western Reserve University Cleveland Ohio USA; ^3^ Department of Neurosciences, Biomedicine and Movement Sciences University of Verona Verona Italy; ^4^ Department of Neuroscience Istituto Superiore di Sanità Rome Italy; ^5^ National CJD Research and Surveillance Unit Centre for Clinical Brain Sciences School of Clinical Sciences University of Edinburgh Edinburgh United Kingdom; ^6^ Sorbonne Université INSERM CNRS UMR 7225 Institut du Cerveau et de la Moelle épinière ICM Paris France; ^7^ Department of Neurology, Memory and Aging Center University of California San Francisco San Francisco California USA

## Abstract

**Objective:**

Real‐time quaking‐induced conversion (RT‐QuIC) assays detect prion‐seeding activity in a variety of human biospecimens, including cerebrospinal fluid and olfactory mucosa swabs. The assay has shown high diagnostic accuracy in patients with prion disorders. Recently, advances in these tests have led to markedly improved diagnostic sensitivity and reduced assay times. Accordingly, an algorithm has been proposed that entails the use of RT‐QuIC analysis of both sample types to diagnose sporadic Creutzfeldt‐Jakob disease with nearly 100% accuracy. Here we present a multi‐center evaluation (ring trial) of the reproducibility of these improved “second generation” RT‐QuIC assays as applied to these diagnostic specimens.

**Methods:**

Cerebrospinal fluid samples were analyzed from subjects with sporadic Creutzfeldt‐Jakob (*n* = 55) or other neurological diseases (*n* = 45) at multiple clinical centers. Olfactory mucosa brushings collected by multiple otolaryngologists were obtained from nine sporadic Creutzfeldt‐Jakob disease cases and 19 controls. These sample sets were initially tested blindly by RT‐QuIC by a coordinating laboratory, recoded, and then sent to five additional testing laboratories for blinded ring trial testing.

**Results:**

Unblinding of the results by a third party indicated 98‐100% concordance between the results obtained by the testing of these cerebrospinal fluid and nasal brushings at the six laboratories.

**Interpretation:**

This second‐generation RT‐QuIC assay is highly transferrable, reproducible, and therefore robust for the diagnosis of sporadic Creutzfeldt‐Jakob disease in clinical practice.

## Introduction

Human prion diseases are fatal, currently untreatable, neurodegenerative diseases of sporadic, genetic, and acquired origin. These disorders arise because of the conformational change of either normal wild type, or mutated, cellular prion protein (PrP^C^) from low β‐sheet monomers^1^, ^2^ into disease‐associated, and usually transmissible, high β‐sheet multimers^3^, ^4^ (generically called prions or PrP^Sc^ ^5^). PrP^Sc^ can propagate exponentially in vivo and in vitro by seeded polymerization, a mechanism in which PrP^C^ monomers are recruited into growing PrP^Sc^ aggregates as refolding occurs.^6^ This seeded polymerization mechanism has been exploited in the development of prion seed amplification assays such as the real‐time quaking‐induced conversion (RT‐QuIC)^7^, ^8^, ^9^ and protein misfolding cyclic amplification PMCA^10^ assays. The RT‐QuIC assays are the most widely used assays for diagnostic purposes and are currently used to diagnose prion diseases in patients using cerebrospinal fluid (CSF) or brushings of the olfactory mucosa (OM). One advantage of RT‐QuIC assays is that, unlike PMCA, they do not generate de novo infectivity.^11^ When applied to CSF specimens, first‐generation RT‐QuIC assays have been reported to have 73‐91% diagnostic sensitivity and close to 100% specificity for sporadic Creutzfeldt‐Jakob disease (sCJD).^9^, ^12^, ^13^, ^14^, ^15^, ^16^, ^17^ The interlab reproducibility of one of the first‐generation RT‐QuIC assays has been evaluated in a multi‐center ring trial, which showed high concordance between analyses performed by 11 different laboratories.^18^ When applied to OM specimens alone, ~97% diagnostic sensitivity and 100% specificity for sCJD have been obtained using RT‐QuIC in two studies involving combined totals of 92 sCJD and 110 non‐CJD subjects.^13^, ^19^

An improved second‐generation RT‐QuIC assay (“Improved QuIC” or “IQ”) has been described more recently with improved sensitivity and assay speed in the analysis of CSF specimens (IQ‐CSF).^15^, ^20^, ^21^, ^22^ IQ‐CSF has provided 92‐97% diagnostic sensitivity and 100% specificity in several largely independent studies when applied to unbiased sets of CSF samples alone.^15^, ^20^, ^21^, ^22^ Direct comparison of the first‐ and second‐generation RT‐QuIC assays on the same samples showed a substantial improvement in diagnostic sensitivity with IQ‐CSF.^15^ The extremely high specificities observed for RT‐QuIC assays of both CSF and OM specimens underpinned a proposal that a positive result from either type of specimen, even if another specimen from the same patient was negative, should be regarded as strong support for an sCJD diagnosis.^19^ Based on this assumption, the combined use of the IQ‐CSF assay for CSF specimens and a first‐generation RT‐QuIC assay for OM specimens improved the accuracy of *intra vitam* sCJD diagnosis to nearly 100% in a study of 61 sCJD and 71 nonprion disease subjects.^19^

Here we report an international multi‐center ring trial evaluation of the reproducibility and robustness of the improved, second‐generation (IQ) RT‐QuIC assays wherein a blinded panel of both CSF (IQ‐CSF) and OM (IQ‐OM) specimens was tested by six laboratories.

## Methods

### Ring trial specimen sites and RT‐QuIC testing centers

**Figure 1:**
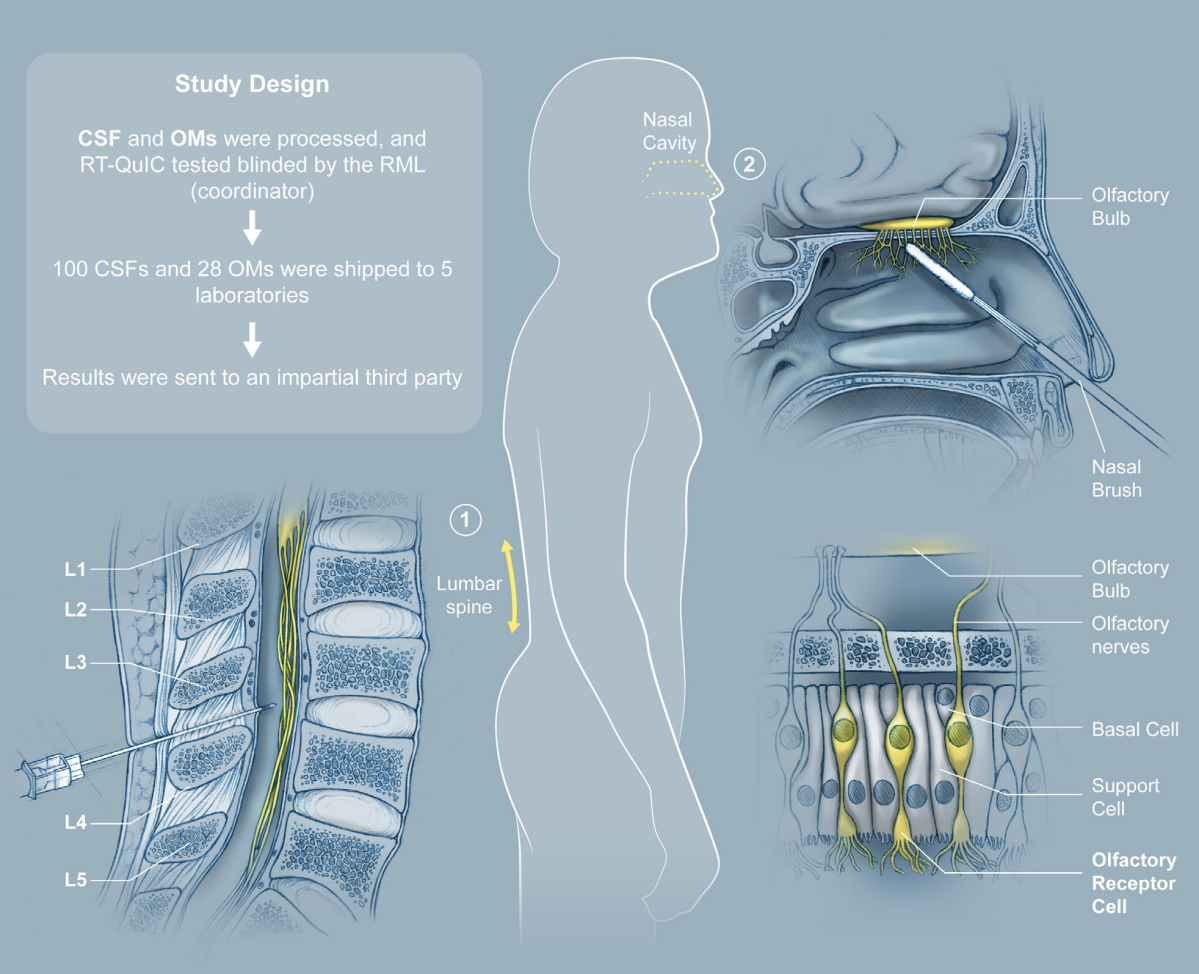
Cerebrospinal fluid and olfactory mucosa ring trial study design.

Four clinical centers provided specimens: University of Verona (UV) and Istituto Superiore di Sanitá (ISS), Italy (combined as Source 1), National Prion Disease Pathology Surveillance Center, US (NPDPSC; Source 2) and University of California‐San Francisco, US (UCSF; Source 3) and six laboratories performed blinded RT‐QuIC testing of CSF and OM samples using the improved RT‐QuIC protocol (Fig. 1) [NIAID Rocky Mountain Laboratories (RML), UV, NPDPSC, ISS, University of Edinburgh (UE), and Sorbonne Université (SU)].

### Ethics statement

CSF sample collections were performed under protocols approved by the ethical committees of the Azienda Ospedaliera Universitaria Integrata of Verona and the ISS (Source 1), the Institutional Review Board at Case Western Reserve University (Source 2), and the Committee on Human Research at UCSF (Source 3). OM sample collections were performed under protocols approved by the Ethical Committee of the University Hospital of Verona (Prot. n. 28917 June 15th, 2012). Written informed consents were obtained from patients or legal representatives and in accordance with the Declaration of Helsinki (1964–2008) and the Additional Protocol on the Convention of Human Rights and Biomedicine Concerning Biomedical Research (2005). All patient data and samples were coded and handled according to NIH guidelines to protect patient identities.

### Patients and clinical evaluations

The patients with rapidly progressive dementia (RPD) and/or suspected sCJD were initially referred to UV, ISS, UCSF, or the NPDPSC for CSF testing. The diagnosis was either neuropathologically confirmed at the NPDPSC Autopsy Coordination Program post hoc (definite CJD), or patients received clinical diagnosis of probable CJD according to WHO^23^, ^24^, ^25^, ^26^ (Source 1 and 2) or UCSF criteria.^27^, ^28^, ^29^ Inclusion criteria also required the availability of CSF samples, date of initial symptoms to ascertain disease duration and, only for definite sCJD, unequivocal classification of Type 1, Type 2, or Type 1‐2 proteinase K‐resistant form of sCJD‐associated PrP^Sc^. OM samples from sCJD, Alzheimer’s disease (AD), Parkinson disease, and healthy voluntary donors (normal controls) were only collected by UV and ISS. The criteria for diagnosis of AD were according to the current National Institute of Aging – Alzheimer's Association guidelines^30^, ^31^ and for other neurological diseases according to recognized diagnostic criteria.

### CJD Classification, brain samples, and *PRNP* gene sequencing

Definite CJD cases were classified on the basis of diagnostic pathology, immunohistochemistry, western blot examination of 2 or 3 brain regions (including frontal, occipital and cerebellum cortices) with mAb 3F4^32^, ^33^, ^34^, ^35^, and in 42/46 samples genotypic analysis of the *PRNP* coding region. For molecular analyses, 200–350 mg of brain tissue samples stored at −80°C after autopsy were used for the unequivocal classification of the proteinase K‐resistant form of sCJD‐associated PrP^Sc^ according to western blot (WB) pattern in Type 1, Type 2, or Type 1‐2.^31^, ^36^ Sixteen anatomical areas were used for histopathological and immunohistochemical classification of prion disease according to NPDPSC’s standard protocols.^33^, ^37^, ^38^ In case of equivocal classification of sCJD subtype between pathology and western blots, cases were classified based on their molecular characteristics of PrP^Sc^ on western blots developed with a panel of Type 1 and Type 2‐specific antibodies as described previously.^33^, ^39^, ^40^ DNA was either extracted from frozen brain tissues (NPDPSC and UCSF) or blood (UV, ISS, and UCSF) and genotypic analysis of the *PRNP* coding region was performed as described.^39^, ^41^, ^42^ Non‐CJD cases did not meet criteria for prion disease.

### CSF sample collection

CSF samples (Tables S1 and S2) were obtained by lumbar puncture from different neurology units in Italy and US (Sources 1 and 2). Samples from Source 3 came only from the UCSF Clinical Translational Science Institute laboratory (CTSI). At the UV and ISS (Source 1), the first 2 mL were used for basic CSF analysis. The next 4 mL was used for 14‐3‐3 and RT‐QuIC testing. Following collection CSFs were at room temperature for an average of 2 h. In case of blood contamination, CSF samples were usually centrifuged at 2,000*g* for 10 min at room temperature before shipping or, if not possible, spun upon arrival in UV or ISS. Samples were usually shipped the same day of collection at 4°C to avoid protein degradation,^43^ and immediately aliquoted and stored at −80°C. When same day shipping was not possible, samples were frozen at either at −20°C or −80°C and sent on dry ice.

At the NPDPSC, the first 2 mL of CSF was discarded and 2–5 mL of CSF, avoiding bloody tap, was collected in polypropylene test tubes; frozen within 20 min of collection, and shipped frozen on dry ice. Upon arrival at NPDPSC, the samples were spun at 2,000*g* for 2 min at 4°C, 1 mL aliquots transferred to polypropylene 1.5 mL tubes with sterile disposable polypropylene pipet tip, and stored until testing at −80°C. For RT‐QuIC testing, the CSF was evaluated for blood contamination and samples above 9600 red blood cells (RBC)/µL corresponding, after freezing and thawing, to the 540 nm absorbance of released hemoglobin equal to 0.389 were excluded from evaluation.^21^

At UCSF, usually the first ~16 mL of CSF was used for clinical diagnostic testing, and aliquots of remaining fluid were stored at CTSI prior to shipment. None of the UCSF samples used were from traumatic taps. Samples were collected through a standard research protocol into a variety of tubes, processed, but not centrifuged, and stored as 250 μL aliquots at −80°C in the UCSF Clinical Translational Science Institute laboratory (CTSI).^44^ Of the 36 CSF samples used in this study, 19 (15 sCJD, four controls) were drawn and processed on the same day with an average handling time of 1.6 ± 0.5 h (all ≤ 2 h) at 4°C; 15 (13 sCJD, two controls) were drawn the day prior to processing and stored at 4°C for less than 24 h; and 2 (sCJD) were drawn at outside hospitals and stored at 4°C for 10 and 20 d, respectively, before being processed. Unlike the samples from the other sources, the UCSF samples were selected from a larger collection of samples from this source after initial blinded IQ‐CSF testing at RML in order to complete the ring trial CSF panel. Of the 30 sCJD samples, three were serial samples from a single sCJD subject.

### OM sample collection

OM swabs were collected from nine sCJD patients who were ultimately classified as definite or probable and 19 nonprion control subjects from six medical centers in Italy (see Table S3). Samples from three sCJD and 19 nonprion subjects were collected at UV, whereas the other six CJDs were from Trieste, Vicenza, San Giovanni Rotondo (FG), and Palermo. Samples collected at UV were processed immediately as previously described.^13^, ^19^ Samples collected outside UV were placed in polypropylene tubes, filled up with saline, sealed, and sent to UV at 4°C within 48 h. Upon arrival at UV, tubes were vortexed and the OM pellets were obtained by centrifugation. Samples were then frozen at −80°C. Only in two cases CSF and OM samples were collected from the same patient (OM‐1 and CSF‐5; OM‐8 and CSF‐1).

### CSF and OM sample distribution by coordinator

After RML received frozen CSF samples, they were thawed at room temperature, aliquoted under sterile conditions and recoded for blinded testing by participating labs. OM samples were thawed at room temperature and processed as described.^13^ Aliquots of the homogenates were recoded for blinded testing and frozen at −80ºC (see Fig. 1). Following an initial blinded RT‐QuIC testing of all the CSF and OM samples by the RML laboratory, the code was broken by the three sources and samples were selected to be shipped frozen to the other five testing laboratories for blinded RT‐QuIC analysis. Each testing laboratory received 100 aliquots of coded individual CSF and 28 aliquots of coded OM homogenate samples from sCJD and nonprion disease patients. RT‐QuIC testing centers were provided with guidelines on the testing methodology and how to classify RT‐QuIC‐positive and ‐negative specimens (see below). The respective results were sent via email to a third independent party (broker) who was not involved in the study and the code was broken.

### RT‐QuIC

Guidelines on assay parameters and criteria for positive/negative determination were provided to each testing center. RT‐QuIC analyses were performed, as previously described.^7^, ^45^, ^46^ Briefly, CSF samples were thawed at room temperature and vortexed immediately before seeding quadruplicate reactions with 20 µL of sample. Each RT‐QuIC reaction mix (prior to adding CSF) was 80 μL of solution^20^ adjusted to give final reaction concentrations of 10 mmol/L phosphate buffer (pH 7.4), 300 mmol/L NaCl, 0.1 mg/mL rPrP^Sen^ Ha rPrP^Sen^ 90–231 (filtered through a 100 kD MWCO filter immediately prior to use), 0.002% sodium dodecyl sulphate (SDS), 10 µmol/L thioflavin T (ThT), and 1 mmol/L ethylenediaminetetraacetic acid tetrasodium salt (EDTA). OM samples were thawed at room temperature and serially diluted in 0.1% SDS/phosphate buffered saline/N_2_ media supplement solution (SDS/PBS/N_2_) such that quadruplicate reactions were seeded with a final tissue dilution of 4 × 10^−3^ and 4 × 10^−4^. Each RT‐QuIC reaction mix was 98 μL^46^ with final reaction concentrations of 10 mmol/L phosphate buffer (pH 7.4), 300 mmol/L NaCl, 0.1 mg/mL rPrP^Sen^ Ha rPrP^Sen^ 90–231 (filtered through a 100 kDa Pall filter), 10 µmol/L thioflavin T (ThT), and 1 mmol/L ethylenediaminetetraacetic acid tetrasodium salt (EDTA). The plates were sealed with a film (Nalgene Nunc International) and incubated at 55°C for >24 h or 50°C for >40 h for CSF and OM samples, respectively. Reactions were cycled through 1‐min shaking (700 rpm, double orbital) and 1‐min of rest, with readings of ThT fluorescence (450ex/480em filter) every 15–45 min. The time cutoff for a positive/negative determination was 24 or 30 h for CSF or OM, respectively. For a sample to be designated positive, the maximum reading for >1 of 4 wells within the designated time cutoff needed to exceed the minimum reading of that well plus 10% of the maximum reading on the plate. Samples with only 2–3 positive replicate reactions out of 4 with an average time to half maximum ThT fluorescence ≥15 h were considered “weak positives” while samples with all four replicate reactions positive and an average lag phase ≤15 h were considered “strong positives”. Testing centers used a BMG FLUOstar OMEGA instrument and recombinant Syrian hamster (90–231) prion protein as the substrate: RML, UE, and SU used substrate generated by RML, whereas UV, ISS, and NPDPSC used their own substrate expressed in *E. coli*, refolded, and purified as described.^45^

## Results

Our ring trial investigated the ability of six testing laboratories to correctly identify CJD‐positive and ‐negative CSF and OM samples. The CSF samples were provided by three different sources: UV and ISS provided 29% of CSFs (Source 1), NPDSC 35% (Source 2), and UCSF the remaining 36% (Source 3, Table 1). CSF samples were analyzed from 55 patients with a final diagnosis of definite (*n* = 46) or probable (*n* = 9) sCJD (Table S1). These included 26 females and 29 males with a mean age of 66.1 years old (SD, 7.2; range 43–80 years) and mean disease duration of 12.0 months (SD, 7.7; range 1–39 months). The distribution of sCJD cases by codon 129 genotype and PrP^Sc^ type is reported in Table S1. Non‐CJD neurological patients in the CSF cohort (*n* = 45; Table S2) included individuals aged 28–90 years old with 30 females and 15 males.

**Table 1:** IQ‐CSF results concordance with diagnosis.

	% Sample set^1^	% Concordance^2^
Source 1	29% (29/100)	100% (174/174)
Source 2	35% (35/100)	100% (210/210)
Source 3	36% (36/100)	97% (210/216)
All Sources	100% (100/100)	99% (594/600)

^1^ Percentage of the total sample set and number of samples (values in parentheses) provided by each source are indicated.

^2^ The percent concordance was obtained as the total number of samples correctly identified by all laboratories divided by the total samples tested by all testing centers. Concordance is reported based on the indicated sources and as overall concordance (all sources).

Nine OM samples (Table S3) were analyzed from patients with a final diagnosis of definite (*n* = 5) or probable (*n* = 4) sCJD. These patients’ ages ranged from 42 to 81 years old (seven females and two males) and had disease durations of 2–23 months. Genotypes at codon 129 included four MM, one MV, two VV, and two not determined. OM samples (Table S4) also were analyzed from non‐CJD individuals ranging from 16–78 years old.

Differences in sample collection and processing were noted between the three sources as described in Methods. All the CSFs and OMs were sent to RML frozen (Fig. 1). Samples were thawed, aliquoted, and frozen at −80°C prior to any testing to ensure that all RT‐QuIC testing centers, including RML, were analyzing samples prepared and stored under identical conditions. To assess the efficiency and reproducibility of the IQ‐CSF and IQ‐OM protocols in multiple laboratories, 100 CSF and 28 OM samples were first tested blinded by RML. IQ testing of the CSFs and OMs, which included samples from weak to strong prion seeding activities (see methods), was shown to have 100% concordance with the diagnosis of CJD/non‐CJD. The samples were then shipped to the other five testing laboratories (Fig. 1) for blinded RT‐QuIC testing.

The concordance with the patients’ diagnoses was 100% for all testing laboratories for CSFs from Sources 1 and 2, and 97% for CSFs from Source 3 (Table 1). None of the testing centers had false positives for the CSF analysis (Table S5). Some false negatives occurred, including two MM2 cases (CSF‐80 and CSF‐91), two MV1/2 (CSF‐74, and CSF‐87), and one MM1/2 (CSF‐78) (Table 2). Only in one case did more than one center have a false negative from the same specimen; two of the three centers had a false negative result for one specimen (CSF‐87) (Table S5). Except for CSF‐78, the CSF samples that were false negative were categorized as weak positives (see Methods) by RML on their initial blinded screening. Samples CSF‐78, CSF‐87, and CSF‐91 could not be retested because of insufficient specimen volume. The two false negatives reported by UV (CSF‐74 and CSF‐80) and SU (CSF‐78 and CSF‐91), and the one false negative reported by ISS (CSF‐87) and UE (CSF‐87) were all from Source 3 (Tables 1 and 2). Overall, samples from this source, which were processed differently from the CSFs provided by Sources 1 and 2 (see Methods), tended to have weaker IQ‐CSF seeding activity in the initial screening done by RML. Regardless, the total concordance between the six testing centers was 99 ± 1% with an overall concordance of 99% for CSFs (Table 3).

**Table 2:** IQ‐CSF concordance by sCJD subtype.

	sCJD Subtype	Individual CSF samples (#)	RT‐QuIC positive/total CSF samples tested	Overall Concordance (%)	Laboratory with discordant result
Definite sCJD	MM1	22	132/132	100	–
	MV1	2	12/12	100	–
	VV2	2	12/12	100	–
	MV2	4	24/24	100	–
	MM2	3^a^	16/18	89	UV, SU
	MM1/2	3^b^	17/18	94	SU
	MV1/2	5^c^	27/30	90	UV, ISS, UE
	VV1/2	1	6/6	100	–
	NA1	2	12/12	100	–
	NA2	2	12/12	100	–
Probable sCJD	MM	2	12/12	100	–
	MV	3	18/18	100	–
	VV	1	6/6	100	–
	NA	3	18/18	100	–
Non‐CJD	–	45	0/270	100	–
Total	–	100	594/600	99	

^a^ Two of three individual CSFs each had one discordant result out of six tests each.

^b^ One of three individual CSFs had one discordant result out of six tests each.

^c^ One of five individual CSFs had one discordant result out of six tests each and one had two discordant results out of six tests each.

**Table 3:** Overall concordance^1^ of CSF testing for each testing laboratory.

	RML	UV	NPDPSC	ISS	UE	SU	
CJD	100% (55/55)	96% (53/55)	100% (55/55)	98% (54/55)	98% (54/55)	96% (53/55)	
Non‐CJD	100% (45/45)	100% (45/45)	100% (45/45)	100% (45/45)	100% (45/45)	100% (45/45)	Total Concordance
Overall	100% (100/100)	98% (98/100)	100/100 (100%)	99% (99/100)	99% (99/100)	98% (98/100)	99% (594/600)

^1^ Concordance (total number of samples that tested correctly out of the total number of samples) for each laboratory as well as the total concordance are displayed.

When testing OM samples, two centers (UV and ISS) had one false negative (Table S6; OM‐8) and one (SU) had a false positive (Table S6; OM‐17). Collectively, the RT‐QuIC analyses of OM samples yielded mean ± standard deviation for concordance of 99 ± 2% (Table 4) between laboratories and an overall concordance of 98%. Our findings so far have indicated strong agreement between the independent blinded assessments by the six testing centers.

**Table 4:** Concordance^1^ of olfactory mucosa testing by testing laboratory.

	RML	UV	NPDPSC	ISS	UE	SU	
CJD	100% (9/9)	89% (8/9)	100% (9/9)	89% (8/9)	100% (9/9)	100% (9/9)	
Non‐CJD	100% (19/19)	100% (19/19)	100% (19/19)	100% (19/19)	100% (19/19)	95% (18/19)	Total Concordance
Overall	100% (28/28)	96% (17/28)	100% (28/28)	96% (27/28)	100% (28/28)	96% (27/28)	98% (165/168)

^1^ Concordance (total number of samples that tested correctly out of the total number of samples) for each laboratory as well as the total concordance are displayed.

## Discussion

With the improved speed and sensitivity of second‐generation RT‐QuIC assays, the need arose to validate the transferability and reproducibility of these new assays. Our multi‐center blinded testing of non‐CJD and sCJD patients showed 99% concordance between all testing laboratories with both CSF and OM specimens (99% for CSF and 98% for OM, respectively). Notably no correlation could be found between the apparent false negatives and the substrate used, whether it was produced at the individual test sites or provided by RML. The fact that in RML’s initial blinded screening, some of the sCJD samples were positive in only a fraction of the replicate wells indicated that the seeding activity in these samples was near the detection limit of the assay; therefore, it is not surprising that some testing centers mis‐identified such sCJD CSF samples as negatives. On the other hand, the testing centers that correctly identified these same samples as positives did not report any false positives, which suggests that false negatives were more likely due to inherent variability of the assay near the detection limit rather than differences in execution between laboratories.

Our CSF cohort included the MM1, MM2, MV1, MV2, and VV2 sCJD subtypes, as defined previously.^31^, ^36^ It also included mixed subtypes MM1/2, MV1/2, and VV1/2, but did not have any VV1 patients. In this cohort, we observed 100% concordance with MM1, MV1, VV2, MV2 and VV1/2 but not with MM2, MM1/2, and MV1/2 sCJD subtypes provided by Source 3. Two previous ring trials^18^ of a first‐generation RT‐QuIC assay for CSF gave 83‐100% concordance for the first ring trial and 100% concordance for the second ring trial. Those assays required 90 h and were applied to a total of 25 CSF samples. In our current trial, the IQ‐RT‐QuIC assays required only 24–30 h and were applied to 100 CSFs and 28 OMs. The six participating laboratories achieved 98‐100% concordance for CSF and 96%–100% concordance for OMs. Moreover, whereas the previous trial was done on CSF samples provided by a single source, our study used CSF samples from three different sources, which better approximated the practical circumstances of diagnostic centers receiving samples from multiple clinicians. Indeed, the CSF samples from Source 3 tended to give weaker RT‐QuIC responses. The reason for this remains unclear, but one systematic difference in sample processing by Source 3 was the lack of a centrifugation step to remove cellular debris that might contain interfering substances. CSF handling and processing times also varied between individual samples from various sources, but there were no systematic differences in timing between Sources 1–3. The samples that were false negative at one or more testing sites were among the subgroup of 15 sCJD samples that were kept at 4°C at least overnight (3 for ~15–18 h and 1 for 20 d) prior to freezing at −80°C, whereas none of the subgroup of 15 sCJD that were processed the same day that CSF was collected (i.e. kept at 4°C ≤ 2 h) gave any false negative results. The effects of freezing and thawing of CSF also remain unclear and may have played a role. The RML and UE groups have noticed losses of RT‐QuIC seeding activity with several freeze‐thaw cycles; however, a study by others concluded that the seeding activity was stable through 16 freeze‐thaw cycles.^14^ The same group also determined that red blood cell contamination greater than 1250 red blood cells/µL had an inhibitory effect, at least when performed by their particular first‐generation RT‐QuIC technique.^14^ This was not an issue, however, with the CSF samples used from the sources in this study. Additional studies are needed to determine how RT‐QuIC results might be affected by centrifugation, storage conditions, and freeze‐thaw cycles.

We also note that some technical difficulties were encountered in the initial phases of the trial. ISS experienced technical problems with their plate reader and was provided with a second aliquot of the samples for repeat testing. Their repeat analysis was performed successfully without further complications. SU went through an optimization phase to resolve problems that were possibly due to the use of materials that were different from the ones recommended previously^45^ (e.g., reagents and plate sealing tapes that failed causing an unusually high incidence of dry wells), the use of unfiltered SDS/PBS for the assay, and a delay in the implementation of the assays due to administrative complications. Subsequently, their testing of a second set of blinded samples was more successful, showing the importance of the quality of materials used in IQ‐RT‐QuIC assays. We note that three of six testing centers in this study had little prior experience with IQ‐RT‐QuIC. The high concordance of their results provided evidence of the interlaboratory transferability of the second‐generation assays. However, the fact that some centers encountered initial technical difficulties illustrated the importance of concordance testing, particularly for laboratories that are inexperienced in RT‐QuIC testing.

Our study also provides the first multi‐site testing of OM samples using the IQ‐OM protocol. OM testing is relatively new so only a limited number of OM samples (28 total) were available for testing in our ring trial. Even so, the six testing laboratories had 96–100% concordance. All the OMs came from Source 1 and were processed for testing by RML. Of the six testing labs, SU had one false positive (OM‐17) and two labs (UV and ISS) had the same false negative sample (OM‐8). Notably, this latter sample was scored as a weak positive (see Methods) by RML. This outcome may also be explained by the inherent variability of the assay near the detection limit. Overall, although several of the centers had never tested OM samples, they implemented the protocol without major issues. Moreover, the OMs were collected at several clinics in Italy by different otolaryngologists, but our analyses showed no evidence that this affected IQ‐OM sample quality. These initial findings suggest that OM samples can be collected, shipped, and tested with reproducible results. Thus, IQ‐OM testing could be considered for use as either a primary assay or a secondary confirmatory step for sCJD diagnosis.

Although further studies are necessary to demonstrate the robustness of these second‐generation assays, this ring trial provides strong validation of their utility and reproducibility as a diagnostic assay for sCJD.

## Conflict of Interest

CDO, BRG, AF, MB, FC, NM, AC, AG, AP, KG, DP, MF, XL, AL, MS, AGH, JGS, and GZ report grants from Alliance Biosecure Foundation, during the conduct of the study.

BC reports grants from Alliance Biosecure and from the Mary Smith Family Foundation, during the conduct of the study. In addition, BC has a patent 2554996 (France, Germany, UK) issued, a patent 2179293: Switzerland, Germany, France, UK, Ireland issued, and a patent 8,216,788: USA issued.

MDG has consulted for 3D Communications, Adept Field Consulting, Advanced Medical Inc., Best Doctors Inc., Second Opinion Inc., Gerson Lehrman Group Inc., Guidepoint Global LLC, InThought Consulting Inc., Market Plus, Trinity Partners LLC, Biohaven Pharmaceuticals, Quest Diagnostics, and various medical‐legal consulting. He has received speaking honoraria for various medical center lectures and from Oakstone publishing. He has received past research support from Alliance Biosecure, CurePSP, the National Institutes of Health, the Tau Consortium, and Quest Diagnostics. Dr. Geschwind serves on the board of directors for San Francisco Bay Area Physicians for Social Responsibility and on the editorial board of Dementia & Neuropsychologia.

MP reports grants from Alliance Biosecure Foundation, during the conduct of the study; personal fees from Ferring Pharmaceuticals, personal fees from CNCCS (Collection of National Chemical Compounds and Screening Center), nonfinancial support from Fondazione Cellule Staminali, outside the submitted work.

SH reports grants from Foundation Alliance Biosecure, during the conduct of the study; grants from MedDay Pharmaceuticals, grants from Institut de Recherche Servier, grants from LFB Biomedicaments, outside the submitted work; In addition, SH has a patent Method For Treating Prion Diseases (PCT/EP 2019/070457) pending.

## Author’s Contributions

C. D. Orrú and B. R. Groveman designed the study, performed sample testing and distribution as well as data analysis and wrote the manuscript. A. Foutz, M. Bongianni, F. Cardone, N. McKenzie, A. Culeux, A. Poleggi, K. Grznarova, D. Perra, M. Fiorini, X. Liu, A. Ladogana, M. Sbriccoli, and A. G. Hughson performed sample testing, data analysis, and edited the manuscript. M. D. Geschwind, M. Pocchiari, J. G. Safar, and G. Zanusso participated in designing the study, selecting the patient cohorts and editing the manuscript. S. Haïk, A. J. Green and B. Caughey designed the study and wrote the manuscript.

## Supporting information


**Table S1.** Patient demographics from the sCJD CSF cohort.
**Table S2.** Patient demographics from the non‐CJD CSF cohort.
**Table S3.** Patient demographics from the sCJD OM cohort.
**Table S4.** Patient demographics from non‐CJD OM cohort.
**Table S5.** Outcome of ring trial blinded RT‐QuIC analysis of individual CSF samples by testing laboratory.
**Table S6.** Outcome of blinded RT‐QuIC analysis of individual olfactory mucosa samples by testing laboratory.
