# Supplementary material for: Ring trial of 2nd generation RT‐QuIC diagnostic tests for sporadic CJD
Source: Ann Clin Transl Neurol. 2020 Nov 13;7(11):2262–71. doi: 10.1002/acn3.51219 (PMC7664259; doi:10.1002/acn3.51219)
Supplement: Supplementary file 1 — Table S1. Patient demographics from the sCJD CSF cohort. Table S2. Patient demographics from the non‐CJD CSF cohort. Table S3. Patient demographics from the sCJD OM cohort. Table S4. Patient demographics from non‐CJD OM cohort. Table S5. Outcome of ring trial blinded RT‐QuIC analysis of individual CSF samples by testing laboratory. Table S6. Outcome of blinded RT‐QuIC analysis of individual olfactory mucosa samples by testing laboratory. [file ACN3-7-2262-s001.docx]

**Supplemental Table 1:** **Patient demographics from the sCJD CSF cohort.**

|  | **Patient #** | **Sex** | **Age** | **Disease duration (months)** | **PMI (h)** | **IHC** | **WB** | **sCJD Subtype** | **Genotype** | **Final diagnosis** |
| --- | --- | --- | --- | --- | --- | --- | --- | --- | --- | --- |
| Source 1 | CSF-1 | F | 68 | 11 | ND | ND | ND | ND | VV | *Probable sCJD* |
|  | CSF-2 | F | 74 | 2 | 24 | ND | + | 1 | NA | **Definite sCJD** |
|  | CSF-3 | F | 76 | 2 | 24 | ND | + | 1 | NA | **Definite sCJD** |
|  | CSF-4 | F | 58 | ND | ND | ND | ND | ND | ND | *Probable sCJD* |
|  | CSF-5 | M | 72 | 8 | 24 | ND | + | 1 | MM | **Definite sCJD** |
|  | CSF-15 | M | 78 | 10 | 48 | ND | + | 2 | NA | **Definite sCJD** |
|  | CSF-16 | M | 70 | 2 | ND | ND | ND | ND | ND | *Probable sCJD* |
|  | CSF-17 | M | 68 | 2 | 48 | ND | + | 1 | MM | **Definite sCJD** |
|  | CSF-18 | M | 78 | 10 | 24 | ND | + | 2 | NA | **Definite sCJD** |
| Source 2 | CSF-30 | F | 70 | 3 | 72 | + | + | 1 | MM | **Definite sCJD** |
|  | CSF-31 | F | 59 | 5 | ND | + | + | 1 | MM | **Definite sCJD** |
|  | CSF-32 | F | 66 | 4 | 48 | + | + | 1 | MM | **Definite sCJD** |
|  | CSF-33 | F | 61 | 5 | 24 | + | + | 1 | MM | **Definite sCJD** |
|  | CSF-37 | M | 68 | ND | ND | + | + | 1 | MM | **Definite sCJD** |
|  | CSF-38 | F | 59 | 2 | ND | + | + | 1 | MM | **Definite sCJD** |
|  | CSF-44 | M | 66 | ND | ND | + | + | 1 | MM | **Definite sCJD** |
|  | CSF-45 | F | 72 | 2 | 48 | + | + | 1 | MM | **Definite sCJD** |
|  | CSF-46 | M | 79 | 2 | 120 | + | + | 1 | MM | **Definite sCJD** |
|  | CSF-47 | F | 64 | 1 | 72 | + | + | 1 | MM | **Definite sCJD** |
|  | CSF-48 | M | 67 | 1 | 72 | + | + | 1 | MM | **Definite sCJD** |
|  | CSF-53 | M | 69 | 2 | 48 | + | + | 1 | MM | **Definite sCJD** |
|  | CSF-54 | F | 69 | 2 | 48 | + | + | 1 | MM | **Definite sCJD** |
|  | CSF-55 | F | 62 | 2 | 0 | + | + | 1 | MM | **Definite sCJD** |
|  | CSF-56 | M | 67 | 2 | ND | + | + | 1 | MM | **Definite sCJD** |
|  | CSF-64 | M | 71 | ND | ND | + | + | 1 | MM | **Definite sCJD** |
| Source 3 | CSF-65 | F | 49 | 9 | 39 | + | + | 2 | VV | **Definite sCJD** |
|  | CSF-66 | M | 54 | 19 | 72 | + | + | 2 | MM | **Definite sCJD** |
|  | CSF-67 | F | 73 | 12 | 96 | + | + | 1 | MV | **Definite sCJD** |
|  | CSF-70 | F | 66 | 4 | 9 | + | + | 1/2 | MV | **Definite sCJD** |
|  | CSF-71 | F | 66 | 9 | 48 | + | + | 2 | MV | **Definite sCJD** |
|  | CSF-74 | F | 68 | 9 | 24 | + | + | 1/2 | MV | **Definite sCJD** |
|  | CSF-75 | M | 65 | 18 | ND | ND | ND | ND | MV | *Probable sCJD* |
|  | CSF-76 | M | 68 | 2 | ND | ND | ND | ND | MM | *Probable sCJD* |
|  | CSF-77 | F | 57 | 12 | ND | ND | ND | ND | MM | *Probable sCJD* |
|  | CSF-78 | F | 60 | 10 | 81 | + | + | 1/2 | MM | **Definite sCJD** |
|  | CSF-79 | M | 72 | 8 | 50 | + | + | 1 | MV | **Definite sCJD** |
|  | CSF-80 | M | 60 | 20 | 84 | + | + | 2 | MM | **Definite sCJD** |
|  | CSF-81 | F | 62 | 4 | 24 | + | + | 1 | MM | **Definite sCJD** |
|  | CSF-82 | M | 63 | 15 | ND | ND | ND | ND | MV | *Probable sCJD* |
|  | CSF-83 | M | 58 | 10 | 36 | + | + | 1/2 | MM | **Definite sCJD** |
|  | CSF-86 | M | 43 | 17 | ND | ND | ND | ND | MV | *Probable sCJD* |
|  | CSF-87 | M | 74 | 25 | 46 | + | + | 1/2 | MV | **Definite sCJD** |
|  | CSF-88 | M | 66 | 13 | 16 | + | + | 1/2 | MV | **Definite sCJD** |
|  | CSF-89 | F | 66 | 3 | 100 | + | + | 1 | MM | **Definite sCJD** |
|  | CSF-90 | M | 66 | 13 | 96 | + | + | 2 | MV | **Definite sCJD** |
|  | CSF-91 | M | 51 | 39 | 170 | + | + | 2 | MM | **Definite sCJD** |
|  | CSF-92 | F | 80 | 15 | 69 | + | + | 1/2 | VV | **Definite sCJD** |
|  | CSF-93 | M | 66 | 13 | 96 | + | + | 2 | MV | **Definite sCJD** |
|  | CSF-94 | F | 69 | 17 | 72 | + | + | 1 | MM | **Definite sCJD** |
|  | CSF-95 | F | 65 | 4 | 24 | + | + | 2 | VV | **Definite sCJD** |
|  | CSF-96 | F | 64 | 2 | 58 | + | + | 1 | MM | **Definite sCJD** |
|  | CSF-97 | M | 71 | 13 | ND | + | + | 1/2 | MV | **Definite sCJD** |
|  | CSF-98 | M | 66 | 13 | 96 | + | + | 2 | MV | **Definite sCJD** |
|  | CSF-99 | F | 70 | 10 | 8 | + | + | 1/2 | MM | **Definite sCJD** |
|  | CSF-100 | F | 66 | 2 | ND | ND | ND | ND | ND | *Probable sCJD* |

F: female; M: male; ND: not done; PMI: post-mortem interval; +: positive; IHC: immunohistochemistry; WB: western blot; MM/VV/MV indicate amino acid at codon 129

**Supplemental Table 2:** **Patient demographics from the non-CJD CSF cohort.**

|  | **Patient #** | **Sex** | **Age** | **Diagnosis** |
| --- | --- | --- | --- | --- |
| Source 1 | CSF-6 | F | 76 | Alzheimer's disease |
|  | CSF-7 | F | 66 | Alzheimer's disease |
|  | CSF-8 | F | 61 | Alzheimer's disease |
|  | CSF-9 | F | 65 | Alzheimer's disease |
|  | CSF-10 | F | 71 | Multiple Sclerosis |
|  | CSF-11 | M | 69 | Encephalitis |
|  | CSF-12 | F | 70 | Alzheimer's disease |
|  | CSF-13 | M | 60 | Alzheimer's disease |
|  | CSF-14 | F | 70 | Alzheimer's disease |
|  | CSF-19 | F | 56 | Multiple sclerosis |
|  | CSF-20 | F | 28 | Multiple sclerosis |
|  | CSF-21 | F | 65 | Amyotrophic lateral sclerosis |
|  | CSF-22 | M | 49 | Amyotrophic lateral sclerosis |
|  | CSF-23 | F | 77 | Amyotrophic lateral sclerosis |
|  | CSF-24 | F | 77 | Amyotrophic lateral sclerosis |
|  | CSF-25 | F | 85 | Alzheimer's disease with Amyloid Angiopathy |
|  | CSF-26 | M | 72 | Amyotrophic lateral sclerosis |
|  | CSF-27 | F | 44 | Multifocal motor neuropathy |
|  | CSF-28 | M | 84 | Encephalitis |
|  | CSF-29 | F | 70 | Alzheimer's disease |
| Source 2 | CSF-34 | M | 84 | Lewy Body variant of Alzheimer's disease |
|  | CSF-35 | M | 29 | Multi-infarct encephalopathy |
|  | CSF-36 | M | 77 | Encephalitis |
|  | CSF-39 | F | 74 | Alzheimer's disease |
|  | CSF-40 | M | 76 | Alzheimer's disease |
|  | CSF-41 | F | 71 | Alzheimer's disease |
|  | CSF-42 | F | 52 | Chronic traumatic encephalopathy |
|  | CSF-43 | F | 72 | Alzheimer's disease |
|  | CSF-49 | F | 67 | Hippocampal sclerosis |
|  | CSF-50 | M | 82 | Alzheimer's disease with cerebral amyloid angiopathy |
|  | CSF-51 | M | 58 | Encephalitis |
|  | CSF-52 | F | 55 | Multi-infarct encephalopathy |
|  | CSF-57 | F | 72 | Alzheimer's disease |
|  | CSF-58 | M | 72 | Leukoencephalopathy |
|  | CSF-59 | F | 57 | Paraneoplastic cerebellar degeneration |
|  | CSF-60 | F | 59 | Lewy Body variant of Alzheimer's disease |
|  | CSF-61 | M | 76 | Leukoencephalopathy |
|  | CSF-62 | M | 40 | Leukoencephalopathy |
|  | CSF-63 | F | 52 | Frontotemporal lobar degeneration, TDP-43 (Type A) |
| Source 3 | CSF-68 | F | 48 | Early onset Alzheimer's disease |
|  | CSF-69 | F | 90 | Anxiety not otherwise specified |
|  | CSF-72 | F | 77 | Not Clear; Catatonia |
|  | CSF-73 | F | 87 | Frontotemporal degeneration |
|  | CSF-84 | F | 63 | Alzheimer's disease |
|  | CSF-85 | M | 70 | Anti-AMPAR |

F: female; M: male.

| **Patient #** | **Sex** | **Age** | **Disease Duration (months)** | **PMI (h)** | **IHC** | **WB** | **sCJD Subtype** | **Genotype** | **Final diagnosis** |
| --- | --- | --- | --- | --- | --- | --- | --- | --- | --- |
| OM-1 | M | 72 | 8 | 24 | ND | + | 1 | MM | **Definite sCJD** |
| OM-6 | M | 64 | 4 | 24 | ND | + | 1 | MM | **Definite sCJD** |
| OM-7 | F | 81 | 3 | 48 | ND | + | 1 | ND | **Definite sCJD** |
| OM-8 | F | 68 | 11 | ND | ND | ND | ND | VV | *Probable sCJD* |
| OM-9 | F | 61 | 3 | ND | ND | ND | ND | ND | *Probable sCJD* |
| OM-10 | F | 55 | 23 | ND | ND | ND | ND | MV | *Probable sCJD* |
| OM-11 | F | 45 | 10 | ND | ND | ND | ND | MM | *Probable sCJD* |
| OM-12 | F | 72 | 3 | 24 | + | + | 2 | VV | **Definite sCJD** |
| OM-13 | F | 58 | 16 | 48 | ND | + | 1/2 | MM | **Definite sCJD** |

**Supplemental Table 3:** **Patient demographics from the sCJD OM cohort.**

F: female; M: male; ND: not done; PMI: post-mortem interval; IHC: immunohistochemistry; +: positive; WB: western blot; MM/VV/MV indicate amino acid at codon 129; All OM samples came from source 1.

**Supplemental Table 4:** **Patient demographics from non-CJD OM cohort.**

| **Patient #** | **Sex** | **Age** | **Diagnosis** |
| --- | --- | --- | --- |
| OM-2 | F | 25 | Normal control |
| OM-3 | F | 52 | Normal control |
| OM-4 | M | 53 | Normal control |
| OM-5 | M | 27 | Normal control |
| OM-14 | M | 76 | Parkinson's disease |
| OM-15 | M | 41 | Normal control |
| OM-16 | M | 71 | Normal control |
| OM-17 | M | 60 | Parkinson's disease |
| OM-18 | F | 16 | Normal control |
| OM-19 | F | 78 | Alzheimer’s disease |
| OM-20 | M | 65 | Alzheimer’s disease |
| OM-21 | M | 66 | Normal control |
| OM-22 | F | 45 | Normal control |
| OM-23 | F | 31 | Normal control |
| OM-24 | M | 56 | Normal control |
| OM-25 | F | 36 | Normal control |
| OM-26 | M | 77 | Normal control |
| OM-27 | F | 65 | Normal control |
| OM-28 | M | 75 | Normal control |

F: female; M: male; All OM samples came from Source 1.

**Supplemental Table 5:** **Outcome of ring trial blinded RT-QuIC analysis of individual CSF samples by testing laboratory.**

|  | **Patient sample** | **RML** | **UV** | **NPDPSC** | **ISS** | **UE** | **SU** | **Diagnosis** |
| --- | --- | --- | --- | --- | --- | --- | --- | --- |
| Source 1 | CSF-1 | +^a^ | + | + | + | + | + | Probable sCJD |
|  | CSF-2 | + | + | + | + | + | + | Definite sCJD |
|  | CSF-3 | + | + | + | + | + | + | Definite sCJD |
|  | CSF-4 | + | + | + | + | + | + | Probable sCJD |
|  | CSF-5 | + | + | + | + | + | + | Definite sCJD |
|  | CSF-6 | -^b^ | - | - | - | - | - | Alzheimer’s disease |
|  | CSF-7 | - | - | - | - | - | - | Alzheimer’s disease |
|  | CSF-8 | - | - | - | - | - | - | Alzheimer’s disease |
|  | CSF-9 | - | - | - | - | - | - | Alzheimer’s disease |
|  | CSF-10 | - | - | - | - | - | - | Multiple Sclerosis |
|  | CSF-11 | - | - | - | - | - | - | Encephalitis |
|  | CSF-12 | - | - | - | - | - | - | Alzheimer’s disease |
|  | CSF-13 | - | - | - | - | - | - | Alzheimer’s disease |
|  | CSF-14 | - | - | - | - | - | - | Alzheimer’s disease |
|  | CSF-15 | + | + | + | + | + | + | Definite sCJD |
|  | CSF-16 | + | + | + | + | + | + | Probable sCJD |
|  | CSF-17 | + | + | + | + | + | + | Definite sCJD |
|  | CSF-18 | + | + | + | + | + | + | Definite sCJD |
|  | CSF-19 | - | - | - | - | - | - | Multiple sclerosis |
|  | CSF-20 | - | - | - | - | - | - | Multiple sclerosis |
|  | CSF-21 | - | - | - | - | - | - | Amyotrophic lateral sclerosis |
|  | CSF-22 | - | - | - | - | - | - | Amyotrophic lateral sclerosis |
|  | CSF-23 | - | - | - | - | - | - | Amyotrophic lateral sclerosis |
|  | CSF-24 | - | - | - | - | - | - | Amyotrophic lateral sclerosis |
|  | CSF-25 | - | - | - | - | - | - | Alzheimer’s disease with Amyloid Angiopathy |
|  | CSF-26 | - | - | - | - | - | - | Amyotrophic lateral sclerosis |
|  | CSF-27 | - | - | - | - | - | - | Multifocal motor neuropathy |
|  | CSF-28 | - | - | - | - | - | - | Encephalitis |
|  | CSF-29 | - | - | - | - | - | - | Alzheimer’s disease |
| Source 2 | CSF-30 | + | + | + | + | + | + | Definite sCJD |
|  | CSF-31 | + | + | + | + | + | + | Definite sCJD |
|  | CSF-32 | + | + | + | + | + | + | Definite sCJD |
|  | CSF-33 | + | + | + | + | + | + | Definite sCJD |
|  | CSF-34 | - | - | - | - | - | - | Lewy body variant of Alzheimer’s disease |
|  | CSF-35 | - | - | - | - | - | - | Multi-infarct encephalopathy |
|  | CSF-36 | - | - | - | - | - | - | Encephalitis |
|  | CSF-37 | + | + | + | + | + | + | Definite sCJD |
|  | CSF-38 | + | + | + | + | + | + | Definite sCJD |
|  | CSF-39 | - | - | - | - | - | - | Alzheimer’s disease |
|  | CSF-40 | - | - | - | - | - | - | Alzheimer’s disease |
|  | CSF-41 | - | - | - | - | - | - | Alzheimer’s disease |
|  | CSF-42 | - | - | - | - | - | - | Chronic traumatic encephalopathy |
|  | CSF-43 | - | - | - | - | - | - | Alzheimer’s disease |
|  | CSF-44 | + | + | + | + | + | + | Definite sCJD |
|  | CSF-45 | + | + | + | + | + | + | Definite sCJD |
|  | CSF-46 | + | + | + | + | + | + | Definite sCJD |
|  | CSF-47 | + | + | + | + | + | + | Definite sCJD |
|  | CSF-48 | + | + | + | + | + | + | Definite sCJD |
|  | CSF-49 | - | - | - | - | - | - | Hippocampal sclerosis |
|  | CSF-50 | - | - | - | - | - | - | AD with CAA |
|  | CSF-51 | - | - | - | - | - | - | Encephalitis |
|  | CSF-52 | - | - | - | - | - | - | Multi-infarct encephalopathy |
|  | CSF-53 | + | + | + | + | + | + | Definite sCJD |
|  | CSF-54 | + | + | + | + | + | + | Definite sCJD |
|  | CSF-55 | + | + | + | + | + | + | Definite sCJD |
|  | CSF-56 | + | + | + | + | + | + | Definite sCJD |
|  | CSF-57 | - | - | - | - | - | - | Alzheimer's disease |
|  | CSF-58 | - | - | - | - | - | - | Leukoencephalopathy |
|  | CSF-59 | - | - | - | - | - | - | Paraneoplastic cerebellar degeneration |
|  | CSF-60 | - | - | - | - | - | - | Lewy body variant of Alzheimer’s’ disease |
|  | CSF-61 | - | - | - | - | - | - | Leukoencephalopathy |
|  | CSF-62 | - | - | - | - | - | - | Leukoencephalopathy |
|  | CSF-63 | - | - | - | - | - | - | Frontotemporal lobar degeneration, TDP-43 (Type A) |
|  | CSF-64 | + | + | + | + | + | + | Definite sCJD |
| Source 3 | CSF-65 | + | + | + | + | + | + | Definite sCJD |
|  | CSF-66 | + | + | + | + | + | + | Definite sCJD |
|  | CSF-67 | + | + | + | + | + | + | Definite sCJD |
|  | CSF-68 | - | - | - | - | - | - | Early-onset Alzheimer's disease |
|  | CSF-69 | - | - | - | - | - | - | Anxiety not otherwise specified. |
|  | CSF-70 | + | + | + | + | + | + | Definite sCJD |
|  | CSF-71 | + | + | + | + | + | + | Definite sCJD |
|  | CSF-72 | - | - | - | - | - | - | Not Clear; Catatonia |
|  | CSF-73 | - | - | - | - | - | - | Frontotemporal dementia |
|  | CSF-74 | + | - | + | + | + | + | Definite sCJD |
|  | CSF-75 | + | + | + | + | + | + | Probable sCJD |
|  | CSF-76 | + | + | + | + | + | + | Probable sCJD |
|  | CSF-77 | + | + | + | + | + | + | Probable sCJD |
|  | CSF-78 | + | + | + | + | + | -* | Definite sCJD |
|  | CSF-79 | + | + | + | + | + | + | Definite sCJD |
|  | CSF-80 | + | - | + | + | + | + | Definite sCJD |
|  | CSF-81 | + | + | + | + | + | + | Definite sCJD |
|  | CSF-82 | + | + | + | + | + | + | Probable sCJD |
|  | CSF-83 | + | + | + | + | + | + | Definite sCJD |
|  | CSF-84 | - | - | - | - | - | - | Alzheimer’s disease |
|  | CSF-85 | - | - | - | - | - | - | Anti-AMPAR encephalitis |
|  | CSF-86 | + | + | + | + | + | + | Probable sCJD |
|  | CSF-87 | + | + | + | -* | -* | + | Definite sCJD |
|  | CSF-88 | + | + | + | + | + | + | Definite sCJD |
|  | CSF-89 | + | + | + | + | + | + | Definite sCJD |
|  | CSF-90 | + | + | + | + | + | + | Definite sCJD |
|  | CSF-91 | + | + | + | + | + | -* | Definite sCJD |
|  | CSF-92 | + | + | + | + | + | + | Definite sCJD |
|  | CSF-93 | + | + | + | + | + | + | Definite sCJD |
|  | CSF-94 | + | + | + | + | + | + | Definite sCJD |
|  | CSF-95 | + | + | + | + | + | + | Definite sCJD |
|  | CSF-96 | + | + | + | + | + | + | Definite sCJD |
|  | CSF-97 | + | + | + | + | + | + | Definite sCJD |
|  | CSF-98 | + | + | + | + | + | + | Definite sCJD |
|  | CSF-99 | + | + | + | + | + | + | Definite sCJD |
|  | CSF-100 | + | + | + | + | + | + | Probable sCJD |

^a^Samples were identified as positive (+; orange) when at least 2 out of the 4 replicate reactions had ThT fluorescence above our set threshold (see methods). ^b^Samples that had zero positive wells out of the 4 replicate reactions were scored as negative (-; Blue). * Sample that could not be re-tested because no longer available. Anti-AMPAR: anti-α-amino-3-hydroxy-5-methyl-4-isoxazolepropionic acid

**Supplemental Table 6:** **Outcome of blinded RT-QuIC analysis of individual olfactory mucosa samples by testing laboratory.**

| **Patient sample** | **RML** | **UV** | **NPDPSC** | **ISS** | **UE** | **SU** | **Di Diagnosisagnosis** |
| --- | --- | --- | --- | --- | --- | --- | --- |
| OM-1 | +^a^ | + | + | + | + | + | Definite sCJD |
| OM-2 | -^b^ | - | - | - | - | - | Normal control |
| OM-3 | - | - | - | - | - | - | Normal control |
| OM-4 | - | - | - | - | - | - | Normal control |
| OM-5 | - | - | - | - | - | - | Normal control |
| OM-6 | + | + | + | + | + | + | Definite sCJD |
| OM-7 | + | + | + | + | + | + | Definite sCJD |
| OM-8 | + | - | + | - | + | + | Probable sCJD |
| OM-9 | + | + | + | + | + | + | Probable sCJD |
| OM-10 | + | + | + | + | + | + | Probable sCJD |
| OM-11 | + | + | + | + | + | + | Probable sCJD |
| OM-12 | + | + | + | + | + | + | Definite sCJD |
| OM-13 | + | + | + | + | + | + | Definite sCJD |
| OM-14 | - | - | - | - | - | - | Parkinson’s disease |
| OM-15 | - | - | - | - | - | - | Normal control |
| OM-16 | - | - | - | - | - | - | Normal control |
| OM-17 | - | - | - | - | - | + | Parkinson’s disease |
| OM-18 | - | - | - | - | - | - | Normal control |
| OM-19 | - | - | - | - | - | - | Alzheimer’s disease |
| OM-20 | - | - | - | - | - | - | Alzheimer’s disease |
| OM-21 | - | - | - | - | - | - | Normal control |
| OM-22 | - | - | - | - | - | - | Normal control |
| OM-23 | - | - | - | - | - | - | Normal control |
| OM-24 | - | - | - | - | - | - | Normal control |
| OM-25 | - | - | - | - | - | - | Normal control |
| OM-26 | - | - | - | - | - | - | Normal control |
| OM-27 | - | - | - | - | - | - | Normal control |
| OM-28 | - | - | - | - | - | - | Normal control |

^a^Samples were identified as positive (+; orange) when at least 2 out of the 4 replicate reactions had Thioflavin T fluorescence above our set threshold (see methods). ^b^Samples that had zero positive wells out of the 4 replicate reactions were scored as negative (-; Blue).
